# The Use of Artificial Intelligence in Caries Detection: A Review

**DOI:** 10.3390/bioengineering11090936

**Published:** 2024-09-18

**Authors:** Khalifa S. Al-Khalifa, Walaa Magdy Ahmed, Amr Ahmed Azhari, Masoumah Qaw, Rasha Alsheikh, Fatema Alqudaihi, Amal Alfaraj

**Affiliations:** 1 Department of Preventive Dental Sciences, College of Dentistry, Imam Abdulrahman Bin Faisal University, Dammam 31441, Saudi Arabia; 2 Department of Restorative Dentistry, Faculty of Dentistry, King Abdulaziz University, Jeddah 21589, Saudi Arabia; wmahmed@kau.edu.sa (W.M.A.); aaaazhari@kau.edu.sa (A.A.A.); 3 Department of Restorative Dental Sciences, College of Dentistry, Imam Abdulrahman Bin Faisal University, Dammam 31441, Saudi Arabia; mssqaw@iau.edu.sa (M.Q.); ralsheikh@iau.edu.sa (R.A.); 4 Department of Restorative Dentistry, Khobar Dental Complex, Eastern Health Cluster, Dammam 32253, Saudi Arabia; fali-alqudaihi@moh.gov.sa; 5 Department of Prosthodontics and Dental Implantology, College of Dentistry, King Faisal University, Al-Ahsa 31982, Saudi Arabia; asalfaraj@kfu.edu.sa

**Keywords:** dental caries, artificial intelligence, detection, diagnosis, treatment planning

## Abstract

Advancements in artificial intelligence (AI) have significantly impacted the field of dentistry, particularly in diagnostic imaging for caries detection. This review critically examines the current state of AI applications in caries detection, focusing on the performance and accuracy of various AI techniques. We evaluated 40 studies from the past 23 years, carefully selected for their relevance and quality. Our analysis highlights the potential of AI, especially convolutional neural networks (CNNs), to improve diagnostic accuracy and efficiency in detecting dental caries. The findings underscore the transformative potential of AI in clinical dental practice.

## 1. Introduction

Dental caries is a chronic, infectious disease that results in the localized destruction or demineralization of dental hard tissues caused by acids released from bacterial fermentation of dietary carbohydrates [1]. It is a multifactorial disease that is affected by factors such as microbial composition, salivary flow and composition, fluoride exposure, the intake of dietary sugars, and the preventive behavior of an individual to maintain oral hygiene [2]. This disease is reversible in its early stages, and, if ignored, it may cause irreversible damage to the tooth. Thus, early diagnosis, monitoring, and prompt intervention are essential to limit further damage to tooth surfaces [3].

Many diagnostic tests have been developed to detect the disease. Dental practitioners are recommended to use a combination of several diagnostic tests to achieve accurate results, enabling efficient treatment planning [4]. For instance, with early caries detection, dentists can intervene and turn an active lesion into an inactive one using minimally invasive procedures such as topical fluoride applications, sealants, and preventive resin restorations to restore early carious lesions [5,6].

Traditionally, visual–tactile examination along with radiography are commonly used methods for caries detection. These tests evaluate the caries status of an individual based on subjective signs such as the color, hardness, and translucency of the lesion. However, these conventional diagnostic methods are known to have low sensitivity, specificity, and reproducibility and cannot even determine the activity or progression of carious lesions [7,8,9].

Meanwhile, several advanced diagnostic methods such as fiber-optic transillumination, quantitative light-induced fluorescence (QLF), laser fluorescence, electrical conductance measurements, digital radiography, and optical coherence tomography (OTC) have been introduced, which give more accurate information about carious lesions. These diagnostic tools work mainly based on principles of transillumination, visible spectrum and laser fluorescence, electric resistance, sensor technology, and infrared radiation [10]. Besides the detection of early carious lesions, these devices provide additional information on the lesion’s extension, specific location, and bacterial activity. Some may even (e.g., laser fluorescence) detect demineralization and remineralization cycles [3]. However, most of these tests have low specificity, which increases the risk of false-positive results during caries detection [11]. Moreover, the high installation cost of most of these devices makes them less feasible in clinical practice [12,13]. Research suggests that this technique displays limited sensitivity due to superimpositions and exposure contrasts. Due to this, more than every second carious lesion remains undetected [14]. Presently, to overcome the limited sensitivity issue of caries detection devices, the use of artificial intelligence (AI) in caries detection has been considered.

In recent times, the introduction of AI in the field of dentistry has played a quintessential role in improving diagnostic accuracy and the standard of care. In the field of medicine, convolutional neural networks (CNNs) are being utilized to evaluate medical imagery [15]. Similarly, AI has been employed in the field of dentistry to analyze dental images. This aids in tooth classification, restoration segmentation, or the diagnosis of normal or pathological structures in photographs, radiographs, transillumination, or photographic imagery and the prediction of treatment outcomes [16]. An image analysis using CNN has been reported to assist clinicians in making reliable and accurate diagnoses. Thus, for the early and accurate diagnosis of dental caries, the use of AI will gradually turn into a new norm. Research studies by Cantu et al. and Mertens et al. have reported higher diagnostic accuracies compared to individual dentists in detecting dental caries [16,17]. However, to date, only a limited amount of literature is available on the use of AI in the diagnosis of early dental caries. Below, we state and explain the AI technologies used in caries detection, the datasets and annotations for AI caries detection, and the performance evaluation of the AI models.

### 1.1. AI Techniques for Caries Detection

The diagnosis of dental caries using visual examination is the most commonly used method in caries detection. This is the preferred method compared to other methods, as it is easy to perform and can achieve satisfactory accuracy [18]. However, research suggests that there are frequently observed situations in clinical practice where different dentists make contradictory diagnoses, resulting in subjective variability in traditional methods used in caries detection. To overcome this, the use of AI has been considered. Presently, three AI-based techniques have been proposed for caries detection. These include image-based caries detection using AI, AI-assisted caries-risk assessment, and the Integration of AI with Computer-Aided Diagnosis (CAD) systems.

#### 1.1.1. Image-Based Caries Detection Using AI

AI techniques have revolutionized the field of dental radiographic analysis. Among these, several key methodologies are utilized.

- Artificial neural networks (ANNs): ANNs are computational models inspired by the human brain’s neural networks. They consist of interconnected nodes (neurons) that process information in layers. In dental caries detection, ANNs analyze patterns in radiographic images to identify carious lesions. They have been shown to achieve high accuracy, making them valuable tools for dental diagnostics [19,20].
- Convolutional Neural Networks (CNNs): CNNs are a type of deep learning model specifically designed for image processing. They use convolutional layers to automatically extract features from images, making them highly effective for analyzing dental radiographs [21]. CNNs have been widely used in caries detection due to their ability to accurately identify and classify carious lesions, particularly in subtle areas that traditional methods may miss. They are highly effective in image and video analyses, natural language processing, and various other domains requiring pattern recognition [22]. These networks excel in identifying local patterns and distinctive features in images through several layers of convolutional and pooling processes, enabling the differentiation between carious and non-carious lesions [23,24].
- Deep Convolutional Neural Networks (DCNNs): DCNNs are advanced forms of CNNs that use multiple layers of convolutional and pooling operations to capture complex patterns in images. These models have been applied to various dental imaging modalities, such as bitewing and panoramic radiographs, to detect and diagnose dental caries. DCNNs have demonstrated high accuracy and reliability, making them a preferred choice for many researchers [25,26].
- Machine Learning (ML) Algorithms: Beyond neural networks, other ML algorithms such as random forest, Gradient Boosting, and Support Vector Machines (SVMs) have been used for caries detection. These algorithms analyze large datasets to identify patterns and predict the presence of carious lesions. ML models are valuable for their ability to handle diverse data types and provide robust predictions [27,28].
- Image Processing Techniques: Image processing techniques are crucial in AI-based caries detection. These techniques involve enhancing image quality, segmenting regions of interest, and extracting relevant features. Methods such as noise reduction, contrast enhancement, and edge detection are commonly used to preprocess dental images before AI model analysis. An improved image quality enables more accurate and reliable caries detection.

In addition to CNNs, recurrent neural networks (RNNs) and long short-term memory (LSTM) networks can be employed for caries detection and for monitoring its progression, though they are less commonly used compared to CNNs [29]. For instance, Cantu et al. reported that a CNN model outperformed dentists with 3–14 years of experience in diagnosing initial carious lesions [17]. Similarly, Devito et al. found promising results using an Artificial Neural Network (ANN) model for proximal caries diagnosis with bitewing radiographs [20]. Another study by Hung et al., using AI technology for predicting root caries, showed good results [27]. Furthermore, Ekert et al. showed that CNNs are effective in identifying apical lesions (ALs) in panoramic dental radiographs [30]. However, the detection of caries in teeth with a complex morphology, such as molars, remains challenging for these models. For instance, a study on detecting caries using a CNN model demonstrated better performance of the model in detecting caries in premolars compared to molars, which have a complex morphology [26]. Another limitation is that current research on automated caries detection focuses solely on images, neglecting patient history and clinical examination findings that dentists typically consider during diagnosis [31].

#### 1.1.2. AI-Assisted Caries-Risk Assessment

The shift towards preventive and minimally invasive dentistry has emphasized the importance of identifying and mitigating risk factors contributing to caries progression. Factors such as oral hygiene practices, dietary habits, one’s socio-economic status, the utilization of dental care services, and one’s attitude towards oral health, are crucial in determining the risk of developing caries [32]. ML models have been developed to predict the risk of root caries based on demographic and lifestyle variables, serving as supplementary tools alongside clinical assessments. These models analyze large datasets, uncovering variables that might not be typically considered [32].

For example, Zanella-Calzada et al. published a study on an AI-based model assessing the dietary and demographic factors that affect dental caries, achieving good accuracy in categorizing individuals with or without caries [31]. Hung M et al.’s AI-based ML model for the diagnostic prediction of root caries reported high accuracy (97.1%), precision (95.1%), and sensitivity (99.6%) [27]. Similarly, Pang et al. introduced an AI-based ML model that predicts the risk of caries based on environmental and genetic factors, though the heterogeneity in this dataset presents a limitation [33].

#### 1.1.3. Integration of AI with Computer-Aided Diagnosis (CAD) Systems

Computer-aided detection and diagnosis (CAD) systems are computer applications that aid in the diagnosis of the disease without any biased opinions. This system utilizes the most advanced image processing techniques combined with AI and ML to diagnose lesions. With the exponential rise in DL algorithms in image-based applications, CAD systems are now being integrated with DL to enhance the existing capabilities in caries diagnosis. This system has the ability to go beyond human memory and diagnose pathological changes [34].

### 1.2. Datasets and Annotations for AI in Caries Detection

Both ML and DL are considered effective methods for diagnosing and predicting the risk of dental caries. The success of these applications heavily relies on the availability of suitable and extensively annotated datasets [35]. Annotated datasets allow for the transfer of human knowledge to AI models by assigning predefined labels, enhancing the models’ ability to accurately interpret data such as images. An increased availability of annotated data improves the model’s performance, precision, and reliability [36].

In caries detection, several specific datasets are utilized, each containing diverse and annotated dental images to train, test, and assess AI models. Examples of such datasets include the HUNT4 Oral Health Study Dataset, containing 13,887 bitewings. It was annotated by six different experts, providing a rich resource for training AI models in detecting various dental conditions, under the AI-Dentify project [37]. Another large dataset is available via the AI-HUB platform, containing 156,965 panoramic and periapical radiographs. This was used to train an AI model by Lee e al. to identify different types of dental implant systems in low-quality and distorted dental radiographs [38].

When collecting data, it is crucial to ensure diversity within the dataset to train AI models effectively across various scenarios, preventing overfitting or underperformance. These diverse datasets enable AI models to achieve optimal performance, represented as Ground Truth (GT), ensuring reliable and accurate caries detection [39].

### 1.3. Performance Evaluation of AI Models

To evaluate the performance of AI models in caries detection, various metrics are used to assess the accuracy and reliability of their predictions. These metrics help determine how well the model distinguishes between carious and non-carious cases [40].

- Sensitivity: This includes the percentage of actual positive cases that are identified as positives, as opposed to those classified as false negatives. The formula used for sensitivity is

TP/(TP+FN)

- Specificity: This includes the percentage of actual negatives that are correctly identified by the model. The formula for specificity is

TN/(TN+FP)

- Precision: This includes the percentage of positive cases that are true positives, as opposed to false positives. The formula used for precision is

TP/(TP+FP)

- F1 score: This provides an average of the sensitivity and precision values. It is calculated as

2×(Precision×Sensitivity)/(Precision+Sensitivity)

- Receiver Operating Characteristic (ROC) curve analysis: The ROC curve is a graphical representation that illustrates the model’s performance by plotting the true-positive rate (sensitivity) against the false-positive rate (1—specificity). Analyzing the ROC curve helps in selecting the optimal threshold that balances accurate and inaccurate predictions.

In this review, we aim to provide an overview of AI algorithms used in caries detection and discuss their clinical implications. We will also address the challenges, limitations, ethical considerations, and future directions in the application of AI for dental caries diagnosis.

## 2. Materials and Methods

To conduct this review, we formulated the following question: “What are the AI algorithms used in caries detection, diagnosis, and prediction, their clinical implications, challenges, limitations, ethical considerations, and future implications?”.

### 2.1. Selection of Resources

A comprehensive search was conducted to retrieve full-length articles. Both electronic and manual searches were performed across various journals. The data required for this review were obtained through a two-stage selection process. In the first stage, articles were selected based on their titles and abstracts in relation to the research topic. The preliminary search yielded 1300 articles. After removing 600 duplicates, 700 articles were passed to the second stage, where inclusion and exclusion criteria were applied.

### 2.2. Eligibility Criteria

- Inclusion criteria
  1. Articles focusing on artificial intelligence applied in caries detection.
  2. Articles with measurable or predictive outcomes to enable quantification.
  3. Articles that properly mentioned the datasets used in assessing the model.
- Exclusion criteria
  1. Articles not written in English.
  2. Unpublished articles that could not be accessed.
  3. Articles with only abstracts and not full texts.

### 2.3. Sources of Data

The literature identification and selection were performed through a comprehensive search of related articles in electronic databases, including Cochrane, Embase, Google Scholar, MEDLINE, PubMed, Saudi Digital Library, Scopus, and Web of Science. The search covered publications from January 2000 to December 2023, using keywords such as AI (artificial intelligence) in dentistry, Artificial Neural Networks (ANNs), Computer-Aided Diagnosis (CAD), convolutional neural networks (CNNs), Deep Learning (DL), and Machine Learning (ML). The search was based on Population, Intervention, Comparison, and Outcome (PICO) elements.

### 2.4. Achieving Results

This review focused on AI technologies used in dentistry for caries prediction, detection, and diagnosis. To summarize the retrieved information, we constructed a table containing details such as the author(s), year of publication, AI algorithm, objectives, factor of study, modality, sample, accuracy/evaluation/significance, comparison, results, and conclusion.

## 3. Results

The exclusion criteria reduced the articles to 49. The journals’ names and the authors’ names were hidden and spread between the authors. A total of 9 more articles were removed due to their focus on dentistry but not specifically on caries. The review eventually carried out a qualitative synthesis on 40 articles that were all fully read. The articles’ years of publishing were considered in order to study AI progressive trends of development and evolution in dentistry over the years. The studies included in this review mainly dealt with the application of AI for the detection, prediction, and diagnosis of dental caries, with many of them having been carried out over the last 15 years (Table 1).

Many of the analyzed studies used convolutional neural networks (CNNs) and Artificial Neural Networks (ANNs). Only two of these studies used different modalities, with one using the Dental Caries Detection Network (DCDNet) and the other applying Deep Convolutional Neural Networks (DCNNs). The main purpose of neural networks was to determine bitewings, panoramic and periapical radiographs, digital panoramic and periapical radiographs, CBCTs, micro-CTs, OCTs, NILTs and TI images, oral and smartphone photographs, and quantitative light-induced fluorescence images in order to support the detection, diagnosis, and prediction of dental caries with higher accuracy and efficiency compared to traditional methods.

## 4. Discussion

The analyzed studies highlight AI’s potential as a promising tool for early and accurate dental caries diagnosis, preventing irreversible tooth structure loss, reducing treatment costs, and decreasing treatment times [67,68]. AI, particularly deep learning, can be used for image processing, automated caries detection, radiographic caries semantic segmentation, and the classification of caries diagnosis and interpretation. Additionally, AI can enhance image quality by reducing noise, boosting resolution, and adding missing features, which aids in accurately locating anatomical and pathological structures [60].

Most studies utilized Artificial Neural Networks (ANNs) and convolutional neural networks (CNNs) for caries detection, diagnosis, and prediction. Notably, two studies employed Deep Convolutional Neural Networks (DCNNs) [26,66]. A summary of AI-based models in dentistry for caries detection, diagnosis, and prediction is provided in Table 1.

### 4.1. Detection and Diagnosis of Caries

Numerous studies evaluated AI models for dental caries detection and diagnosis using various radiographs, including bitewings, panoramic radiographs, oral photographs, and periapical radiographs. Some studies utilized smartphone photographs and near-infrared-light transillumination (NILT) images [16,18,19,20,21,23,25,26,27,28,30,47,48,49,51,53,55,56,60,61,62,63].

Overall, these AI models performed well, with accuracy levels above 92% in most cases. For instance, Geetha et al. reported a 97.1% accuracy in caries diagnosis using an ANN, surpassing traditional methods [47].

Contrarily, Dayi et al. (2023) found that while an AI system based on deep learning effectively detected occlusal and proximal caries, it showed weaker performance in cervical caries detection [66]. Duong et al. achieved a 92.37% prediction accuracy using AI models on smartphone photos and an 87.39% detection accuracy in another model [42,54]. In the two studies where near-infrared-light transillumination (NILT) images were used to automatically detect and localize dental caries, the first, by Casalegno et al. (2019), found the DL approach as having increased the accuracy and speed of caries detection, while the second, by Schwendicke et al. (2020), satisfactorily detected dental caries [23,63].

These findings suggest that AI models can significantly support dentists by providing high-throughput diagnostic assistance, leading to improved patient outcomes. Furthermore, the use of NILT images for caries detection could be beneficial in care homes, rural centers, and schools.

### 4.2. Prediction of Caries

AI’s capability extends beyond diagnosis to predicting disease progression and treatment outcomes, shifting towards a preventive model of care. Studies have summarized the use of AI models for caries prediction, with notable performance and high prediction accuracies. For example, Zaorska et al. reported a 93% prediction accuracy, and Hur et al. achieved an ROC of 0.88 to 0.89 in predicting caries on 2nd molars associated with affected 3rd molars [19,28,33,46,57].

In terms of prediction, the models showed better performance and high prediction accuracies. Zaorska et al. found a prediction accuracy of dental caries of 93%, Park et al.’s model performed favorably in predicting early childhood caries with an AUC of 0.774–0.785, while Hur et al.’s AI model predicted dental caries on 2nd molars associated with affected 3rd molars in panoramic radiographs and CBCT significantly better than other models (with an ROC of 0.88 to 0.89.) [28,46,57]. Furthermore, Pang et al.’s model for the prediction of the risk of caries based on environmental and genetic factors accurately identified patients of a high and very high risk of dental caries, while De Araujo et al.’s model for the prediction and detection of radiation-related caries (RRC) in panoramic radiographs was highly accurate in both the detection and diagnosis of RRC [19,25,33,46]. Another study, by Javed et al., that used Artificial Neural Networks to predict the levels of post-Streptococcus mutans before the excavation of dental caries based on pre-Streptococcus mutans using an iOS App in 45 primary molars with occlusal caries reported a prediction accuracy of close to 100% (99.03%) [44].

AI models thus hold promise for identifying high-risk patients at the community level, aiding in the design of targeted oral hygiene practices and dietary recommendations and assisting clinicians in decision making and preventive treatment planning.

### 4.3. Caries-Risk Assessment

The paradigm in caries management towards prevention and minimum-intervention dentistry has led to the identification and elimination of risk factors that contribute to the progression of the disease. Several factors, such as oral hygiene practices, dietary habits, one’s socio-economic status, the utilization of dental care services, and one’s attitude towards oral health, help determine the risk of developing caries [32]. Early identification of these factors helps prevent the onset of the disease. Various ML models have been created to predict the risk of root caries based on demography and lifestyle variables. The use of ML models may serve as a supplementary tool in the identification of risk factors in addition to the clinician’s perception while predicting future risks of caries. These models analyze large datasets that provide information on variables that would not have been normally considered [32]. Zanella-Calzada et al. published a study on an AI-based model assessing the dietary and demographic components that affect dental caries [31]. Based on dietary and demographic data, this model reported good accuracy in categorizing individuals with or without caries. Another AI-based ML model by Hung M et al. for the diagnostic prediction of root caries reported an accuracy of 97.1%, a precision of 95.1%, and a sensitivity of 99.6% [27]. In addition, Pang et al. reported an AI-based ML model that predicted the risk of caries based on environmental and genetic factors. However, the presence of heterogeneity in the dataset is one of this study’s limitations [33].

### 4.4. Comparative Analysis

Several studies have analyzed the effectiveness of different AI models. Oztekin et al. (2020) detected dental caries using three explainable deep learning models: EfficientNet-B0, DenseNet-121, and ResNet-50 [48]. Although the authors found that all three models similarly identified dental caries with high accuracy and reliability, the ResNet-50 model produced a slightly better performance in comparison to the others. Duong et al. (2021) also compared the detection and classification performance of three models, GoogleNet, ResNet18, and ResNet50, on dental caries through smartphone photographs and found that GoogleNet outperformed both ResNet18 and ResNet50 [42]. Zheng et al. (2021) evaluated and compared three CNN models, VGG19, Inception V3, and ResNet18, for deep dental caries diagnosis and found that ResNet18 displayed good performance among the three [58]. Moran et al. (2021) identified approximal dental caries in bitewing radiographs (with the CNN model Inception) with ResNet as the reference model, and, in comparison to ResNet, this model showed promising outcomes [53].

A number of studies have also evaluated the performance of specific AI models to those of professionals in dentistry. In most studies, AI models performed better than the professionals. Huang et al. (2021), who detected dental caries using the AI models AlexNet, ResNet-152, ResNext-101, VGG-16, and Xception, found out that ResNet-152 CNN models distinguished pathological tooth structures better than clinicians [62]. Cantu et al. compared the efficiency of deep learning models to individual dentists using a convolutional neural network (U-Net) and found that the dentists had an average accuracy of 0.71, while the trained neural network had a precision of 0.80. The results of this study show that, in comparison to dentists, the neural network was more specific (0.83 vs. 0.9) and more sensitive (0.75 vs. 0.36) [17].

Other studies, however, found no significant difference in the performance of professionals and those of AI models. Lian et al. (2021) evaluated the DL technique for dental caries lesion detection (nnU-Net) and classification (DenseNet121) on panoramic radiographs [52]. In this study, the models showed similar results to those of expert dentists. Likewise, a study by Moran et al., evaluating the effectiveness of deep CNN algorithms for detecting and diagnosing dental caries on periapical radiographs using 480 images of teeth, also reported no statistically significant difference between experienced dentists and the CNN algorithm [53].

A study by Lee et al., assessing a CNN model, reported that the deep learning model’s ability in dental caries detection could help dentists make more accurate dental caries diagnoses [26]. However, additional training data are needed. Similarly, Bayrakdar et al. used CNN-based AI algorithms to create models for autonomous caries detection and segmentation [21]. The AI models proved to be more effective than expert observers and helper specialists. This study did, however, note its limitations, including the use of a smaller sample size, a single facility, and training with the same parameters. A recent study by Ahmed et al. intended to evaluate the effectiveness of automated AI models in identifying and classifying dental caries based on the King Abdulaziz University (KAU)-modified (ICDAS) system from dental bitewings. The results of this study validated the high potential for developing an accurate caries detection model that facilitates the diagnosis and classification of dental caries [50].

### 4.5. Clinical Implications

Findings highlight the significant potential of AI technologies in enhancing the detection, diagnosis, and risk assessment of dental caries. AI models, particularly those utilizing convolutional neural networks (CNNs) and Artificial Neural Networks (ANNs), have demonstrated high accuracy, sensitivity, and specificity in various imaging modalities, often outperforming traditional diagnostic methods and even experienced clinicians. The integration of AI into clinical practice could lead to an earlier and more accurate detection of caries, allowing for more timely and effective interventions. Additionally, AI-assisted risk-assessment models can identify individuals at a higher risk of developing caries, enabling personalized preventive strategies and more efficient resource allocations. As AI continues to evolve, its implementation in routine dental care could improve diagnostic consistency, reduce subjective variability, and ultimately enhance patient outcomes. However, the transition to AI-supported dentistry must be managed carefully, addressing ethical considerations, data privacy, and the need for continuous validation against clinical standards.

### 4.6. Challenges and Limitations

AI has promising results in early caries detection with an accuracy equal to or even better than those of dentists. However, it has several limitations. The images must be segmented manually, which takes a significant amount of time. The obtained images must have an adequate size and focus on a small region so that the system can concentrate on the object being studied while covering enough area to include relevant information [43]. The procedure of radiographic assessment through AI involves multiple steps, starting from image acquisition, preprocessing, segmentation, feature extraction, classification, post-processing, and finally validation, and these steps are technique-sensitive, leading to chances of biases and errors being high [69]. In addition, the continuously changing oral environment and the huge discrepancy in the response of patients to oral hygiene instructions could drastically change a patient from a high-caries-risk category to a low-caries-risk category, which is extremely difficult for the AI to predict. Thus, there has to be an interdisciplinary approach between the AI systems and the clinicians to arrive at a proper diagnosis of carious lesions; otherwise, misinterpretations might occur. In addition, these AI applications require large and diverse annotated data to train these models to accurately understand various data available in the form of images, audio, or videos. Moreover, AI-powered caries detection systems cannot replace good oral hygiene practices and regular dental visits. They can only serve as an aid to enhance preventive care.

### 4.7. Prospects and Future Directions

With the rapid advancements in artificial intelligence (AI), the field of dentistry is experiencing a significant transformation, particularly in caries detection.

- The enhancement of detection accuracy: AI algorithms, particularly those utilizing deep learning techniques, can analyze radiographic images with remarkable precision. These algorithms are trained on vast datasets of dental images, enabling them to identify early signs of caries that might be subtle or challenging for the human eye to detect. By learning from a multitude of patterns and anomalies, AI systems can provide highly accurate assessments of carious lesions, which can help in detecting caries at an earlier stage than traditional methods.
- Improved efficiency and reduced time: AI tools can process and analyze dental images much faster than manual methods. This speed reduces the time clinicians spend on diagnosis, allowing them to focus more on patient care and treatment planning. For instance, AI-powered systems can provide instant feedback during radiographic evaluations, streamlining the diagnostic process and enabling quicker decision making.
- A consistent and objective analysis: One of the key advantages of AI in caries detection is its ability to deliver consistent and objective analyses. Unlike human practitioners, AI algorithms do not experience fatigue or variability in performance. This consistency ensures that caries detection remains reliable across different cases and practitioners, minimizing the risk of oversight and improving the overall diagnostic accuracy.
- Enhanced treatment planning: AI systems can integrate data from various sources, including patient history, radiographs, and clinical notes, to provide comprehensive insights into carious lesions. This integration supports more informed treatment planning by offering detailed risk assessments and predictive analytics. As a result, clinicians can tailor treatment plans to the specific needs of each patient, potentially improving outcomes and reducing the need for invasive procedures.
- Patient benefits: For patients, AI-driven caries detection can lead to an earlier diagnosis and less-invasive treatments. Early detection often results in less-extensive restorations, preserving more of the natural tooth structure and improving long-term dental health. Moreover, the increased accuracy and efficiency of AI can reduce the frequency of unnecessary follow-up appointments and procedures, enhancing the overall patient experience.
- Training and support for clinicians: AI tools can also serve as valuable educational resources for dental professionals. By providing real-time feedback and analyses, AI systems can help clinicians refine their diagnostic skills and stay updated with the latest advancements in caries detection. This continuous learning support contributes to professional development and ensures that practitioners can deliver the highest standard of care.

AI has the potential to revolutionize caries detection in dentistry by enhancing accuracy, improving efficiency, and providing objective and consistent analyses. The benefits extend to both dental professionals and patients, promising a more effective, precise, and patient-centered approach to managing carious lesions. As AI technology continues to evolve, its integration into dental practice will likely lead to even more advanced and beneficial applications, further transforming the field of dentistry.

To fully realize the potential of AI in caries detection, several research areas warrant further exploration:

- Algorithm improvements: Continued research is needed to enhance the accuracy and reliability of AI algorithms. This includes developing more robust models that can handle diverse patient populations and varying image qualities.
- Integrations with other diagnostic tools: investigating how AI can be integrated with other diagnostic modalities, such as optical coherence tomography or intraoral scanners, could provide a more comprehensive approach to caries detection.
- Long-term-impact studies: research should focus on the long-term effects of AI integration on patient outcomes, treatment efficacy, and overall dental practice efficiency.

### 4.8. Ethical and Regulatory Considerations

AI is a revolutionary system, but along with that comes the concern of patient data safety and privacy. AI models require vast amounts of patients’ sensitive information for training and testing purposes. This hampers the privacy and security of patients’ personal information, which is a great concern, as it can be misused. Educating the patient about the usage of their information for AI training can be a difficult task, and obtaining informed consent for the same is also challenging [70]. A significant legal issue in AI-based dental caries diagnosis is determining who should be held responsible in the case of misdiagnosis or treatment failure: the dentist, the AI developer, or both. As AI systems become more autonomous, assigning liability becomes increasingly complex and necessitates legal discussions and potentially new legislative frameworks [69]. Another issue associated with AI dental assistance is liability. If the dental clinic or clinician depends completely on the AI system for diagnosis, then the chances of errors are high. Therefore, the clinician will have to be extra cautious when dealing with information provided by the AI.

### 4.9. Strengths and Limitations of This Review

This review article has several notable strengths. It provides a comprehensive overview of the application of AI in caries detection, integrating findings from multiple studies to present a holistic picture of the current state of the field. By critically analyzing 40 articles published over the last 23 years, this review captures the evolution and advancements in AI technologies within dentistry. Additionally, this review highlights various AI techniques, such as CNNs, and their comparative performance against traditional diagnostic methods, thereby underscoring the potential of AI to revolutionize caries detection. The inclusion of diverse data sources and comprehensive search strategies also minimizes the risk of missing significant studies, ensuring a thorough exploration of the topic.

However, there are also some limitations. Firstly, this review primarily focuses on articles published in English, potentially excluding relevant studies in other languages. This review’s non-systematic approach could introduce a selection bias, possibly omitting studies that do not explicitly mention the targeted AI technologies. Furthermore, this review synthesizes results from a limited number of studies, which might not fully capture the diversity and advancements in AI applications for caries detection. Finally, variations in study designs, datasets, and evaluation metrics among the included studies complicate direct comparisons and generalizations of the findings. A systematic review and meta-analysis on this topic would further highlight the findings of this review.

## 5. Conclusions

AI serves as a promising tool in the diagnosis of dental cavities. The results of this study validate the high potential for developing accurate models for caries detection that facilitate the diagnosis and classification of dental caries. This, along with its rapid processing capabilities, positions AI as a highly effective diagnostic tool within this field.

In addition to convolutional neural networks (CNNs), various AI techniques, such as Artificial Neural Networks (ANNs) and Deep Convolutional Neural Networks (DCNNs), have shown significant potential in dental image analysis and cavity detection. These models offer diverse approaches to improving diagnostic accuracy and efficiency.

As this field progresses and becomes more established, with due regard for regulatory and ethical affairs, AI models can become a modern-day tool for dental image analysis and cavity detection. However, the following question remains: can AI models surpass the diagnostic abilities of dentists in identifying cavities in radiographic images and ultimately lead to improved clinical results for patients? Future research should continue to explore the integration of AI with other emerging technologies to enhance diagnostic capabilities and clinical outcomes.

## Figures and Tables

**Table 1:** Studies in which AI-based models were used in dentistry for the detection, diagnosis, and prediction of caries.

Study	AI Algorithm	Objective	Modality	Sample	Accuracy/Evaluation/Statistical Significance	Key Findings
Karhade et al., 2021 [41]	ANN	Classification for Early Childhood Caries (ECCs) through an automated ML algorithm	Datasets	6040 (5123 for training and 1281 for testing)	0.74 AUC, 0.67 sensitivity and 0.64 PPV.	Results compared with 10 clinical examiners. The ML model had similar performance to that of the reference model.
Duong et al., 2021 [42]	ANN	Automated ML algorithm for the detection of dental caries through smartphone photographs	Photos using a smartphone	620 teeth (validating, 80% and testing, 20%)	92.37% accuracy, 88.1% sensitivity and 96.6% specificity	The results were compared with four trained and calibrated dentists. The model showed great potential for clinical diagnostics with considerable accuracy at minimal expense. Further improvement and verification are required for vector machine support.
Ramos-Gomez et al., 2021 [43]	ANN	ML algorithm for the identification of survey items that predict dental caries (random forest)	Datasets	182 subjects	For caries parent’s age classification (MDG of 0.84; MDA of 1.97), unmet needs (MDG of 0.71; MDA of 2.06). Caries parent’s age prediction (MDG of 2.97; MDA of 4.74), with oral health complications in last 1 year (MDG of 2.20; MDA of 4.04)	The results were compared with two trained dentists. The model demonstrated potential for dental caries screening among children.
Javed et al., 2020 [44]	ANN	Predicting post-Streptococcus mutans prior to the excavation of dental caries based on pre-Streptococcus mutans using an iOS App	Datasets	45 patients	Predicts post-Streptococcus mutans with an efficiency of 0.99033, mean squared error and mean absolute percentage error for testing cases were 0.2341 and 4.967 respectively	A logistic regression model was used. The model efficiently generalized the non-linear relationship between pre- and post-Streptococcus mutans for three different caries excavations. Clinicians can take advantage of the model to efficiently predict post-Streptococcus mutans with the aid of the developed PSm iOS App without needing an internet connection.
Pang et al., 2021 [33]	ANN	Predicting the risk of caries based on environmental and genetic factors (AI-based ML model)	Datasets	953 patients; 633 for training and 320 for testing	0.73 AUC	A logistic regression model was used. The AI model accurately identified patients of a high and very high risk of dental caries. A powerful technique for the identification of patients at a high risk of dental caries at the community level.
Hur et al., 2021 [28]	ANN	Predicting dental caries on 2nd molars associated with affected 3rd molars in panoramic radiographs and CBCT	CBCT images and panoramic radiographs	1321 patients (2642 impacted mandibular 3rd molars; 1850 for training and 792 for testing)	0.88 to 0.89 ROC	The results were compared with a reference standard (single predictors). The model performed significantly better at predicting dental caries than other models. The model could be of great help to clinicians for decision making and preventive treatment on 3rd molars.
De Araujo et al., 2021 [19]	ANN	Predicting and detecting radiation-related caries (RRCs) on panoramic radiographs (AI-based model)	Digital panoramic radiographs	15 head and neck cancer patients	Detection, 98.8% accuracy and 0.9869 AUC; prediction, 99.2% accuracy and 0.9886 AUC	The results were compared with two expert dentists. The model demonstrated high detection and diagnostic accuracy for RRCs. The models could help in designing HNC patients’ preventive dental care.
Wu et al., 2021 [45]	ANN	Identification of oral microbes (caries-related) in cross-sectional mother–child dyads	Datasets	For children dental caries prediction models: 36 plaque and 37 salivary samples. For mother dental caries prediction models: 32 plaque samples	AUCs of 0.78, 0.82, and 0.73, respectively, for the child’s plaque model, for the child’s saliva model, and for the mother’s plaque model	The results were compared with the reference standard. The models attained desirable outcomes for both children and mothers. To fine tune these models in the future, more variables should be considered.
Park et al., 2021 [46]	ANN	Prediction of early childhood caries using ML-based artificial intelligence models (Final model, LightGBM algorithms, random forest, and XGBoost)	Datasets	4195 (2936 for training and 1259 for testing)	AUC of 0.774–0.785	A traditional regression model was used. The AI models demonstrated favorable performance in the prediction of dental caries. The model could be utilized in the identification of a high-risk group and the implementation of preventive treatments.
Geetha et al., 2020 [47]	ANN	Diagnosing dental caries in digital radiographs	Digital radiographs (intraoral digital images)	145 intraoral digital images	97.1% accuracy, a 2.8% false-positive rate, a 0.987 ROC area, and a 0.987 PRC area	The results were compared with an expert dentist. The back-propagation neural network model predicted dental caries more accurately than traditional methods. Datasets of high quality and quantity and improved algorithms may exhibit better outcomes in dental practice.
Hung et al., 2019 [27]	ANN	ML model for diagnosing and predicting root caries	Datasets	7272 training cases and 1818 testing cases	97.1% accuracy, 95.1% precision, 99.6% sensitivity, 94.3% specificity, and 0.997 AUC	Reference models and professional dentists served as comparators. The model had the best performance. It could be implemented for clinical diagnosis and could used by both dental and non-dental professionals. It is based on
Zanella-Calzada et al., 2018 [31]	ANN	Analyzing the demographic and dietary factors determining dental caries and oral health	Datasets	6868 training cases and 2944 testing cases	0.69 accuracy and 0.69 and 0.75 AUC values	National Health and Nutrition Examination Survey Data. High accuracy in diagnosis of caries based on demographic and dietary and variables. This model could be helpful to dentists through the provision of an easy, fast, and free dental caries diagnosis tool.
Devito et al., 2008 [20]	ANN	Diagnosis of proximal DC using an AI-based model	Bitewing radiographs	160 radiographs	0.884 AUC	The results were compared with 25 examiners. This model improved the performance on proximal caries diagnosis. When all examiners were considered, the use of neural networks improved the diagnosis rate by 39.4%.
Oztekin et al., 2023 [48]	CNN	Detecting dental caries using three explainable deep learning models: EfficientNet-B0, DenseNet-121, and ResNet-50	Panoramic radiographs	562 subjects	92% accuracy, 87.33% sensitivity, and a 91.61% F1 score	The results were compared with experienced dentists. All the three models similarly identified DC with high accuracy and reliability. However, the ResNet-50 model produced slightly better performance in comparison to the other two. The heat maps could be used by dentists to reduce miscalculations and to validate the classification of the results.
Wang et al., 2023 [49]	CNN	Automatic dental caries and calculus diagnosis using fluorescence sub-band imaging together with deep learning models	Datasets	For training, 54; for validating, 8; and for testing, 16	Means: 96.82% accuracy, 96.56% sensitivity, 99.22% specificity, and a 96.57% F1 score	The results were compared with reference models. The model performed competitively in comparison to the existing methods. This low-cost, highly accurate, and portable method could potentially be used for caries detection both at home and in the community.
Ahmed et al., 2023 [50]	CNN	Evaluating the effectiveness of automated AI models in identifying and classifying dental caries based on the King Abdulaziz University (KAU)-modified (ICDAS) system from dental bitewings	Bitewing radiographs with a 1876 × 1402 pixel resolution	554 bitewing radiographs for testing	0.55 model mean score and 0.535 mean F1 score for proximal carious lesions, while the segmentation model showed 0.76 sensitivity, 0.87 precision, and a 0.81 F1 score	The results were compared with two experienced dentists. The model outperformed the two experienced dentists in identifying and classifying dental caries. This study validated the potential for developing an accurate caries detection model to expedite the identification of caries, enhancing the decision making of clinicians and improving patients’ quality of care.
Bayrakdar et al., 2022 [21]	CNN	Automated detection and segmentation of caries on bitewing radiographs using DL models (VGG-16 and U-Net)	Digital bitewing radiographs	2325 images on 621 patients (2072 for training, 200 for validating, and 53 for testing)	For the detection of caries, 0.84 sensitivity, 0.81 precision, and 0.84 F-measure rates; for caries segmentation, 0.86 sensitivity, 0.84 precision, and 0.84 F-measure rates	The results were compared with five experts and experienced observers. The models not only accurately detected dental caries but were also beneficial in their segmentation. The models could be helpful in the clinical decision making of clinicians, since they demonstrated superior performance to that of specialists.
Zhang et al., 2022 [51]	CNN	Assessing the performance of ConvNet (a CNN-based model) for detecting dental caries by oral photographs	Oral photographs	3932 photographs (training, 2507; testing, 1125) of 625 subjects	85.65% AUC and 81.90% sensitivity	The results were compared with three certified dentists. The model demonstrated promising outcomes for the detection of dental caries in oral photographs. It is a cost-effective technique for dental caries screening.
Kühnisch et al., 2022 [18]	CNN	Evaluating dental caries detection and categorization of a CNN-based model using oral photographs	Oral photographs	2417 photographs (training, 1891; testing, 479)	92.5% accuracy, 89.6% sensitivity, 94.3% specificity, and 0.64 AUC	The results were compared with a reference model. The model showed considerable accuracy in dental caries detecting using intraoral photographs. The model could potentially be useful in the future.
Lian et al., 2021 [52]	CNN	Identification of caries lesions and categorizing radiographic extensions on panoramic films according to depth (dentin lesions in the outer, middle, or inner third D1/2/3 of the dentin)	Panoramic radiographs	Not stated	0.785 intersection over union (IoU), 0.663 Dice coefficient value, 0.986 accuracy, and 0.821 recall rate	The results were compared with six experienced dentists. Both neural networks and experienced dentists produced the same outcomes. The models ought to be explored for the diagnosis of diseases and planning for treatment.
Moran et al., 2021 [53]	CNN	Evaluating the effectiveness of deep CNN algorithms for detecting and diagnosing dental caries	Periapical radiographs	480 images of teeth	73.3% accuracy	The results were compared with less-experienced dentists. No statistically significant difference between less-experienced dentists and the CNN algorithm was found. This model could help physicians make more accurate dental caries diagnoses.
Duong et al., 2021 [54]	CNN	Detecting and classifying dental caries through smartphone photographs	Photos using a smartphone	587 extracted teeth (for training, 80%; for validating, 10%; and for testing, 10%)	87.39% accuracy, 89.88% sensitivity, and 68.86% specificity	The results were compared with trained dentists. The model showed good accuracy in detecting dental caries. It’s GoogleNet performance was better than those of ResNet18 and ResNet50. Training of the model ought to be performed with both in vivo and vitro images. There is need for the development of a good imaging method for occlusal surfaces.
Askar et al., 2021 [55]	CNN	Detection of white spot lesions by digital camera photographs (DL model)	Digital camera images	2781 labelled teeth of 51 patients	For the detection of any lesions (PPV/NPV), between 0.77 and 0.80; for the detection of fluorotic lesions, 0.67 PPV–0.86 NPV; and for the detection of non-fluorotic lesions, 0.46 PPV–0.93 NPV	The results were compared with a trained dentist. The model demonstrated sufficient accuracy in the detection of white spot lesions, especially fluorosis. For generalizability, there is a need of more datasets.
Chen et al., 2021 [25]	CNN	Dental disease detection on periapical radiographs (DL model)	Digital periapical radiographs	2900 periapical radiographs	Detection of lesions was performed with precision and recalls of 0.5–0.6 at all levels	The results were compared with a trained expert and a reference model. The models could detect dental caries through periapical radiographs. The utilization of these models for lesion detection is best at severe levels. Therefore, there is a need for more training at various levels.
Devlin et al., 2021 [56]	CNN	Detecting dental caries (enamel-only proximal) on bitewing radiographs using AssistDent AI software	Bitewing radiographs	24 patients	In comparison to expert dentists, a high diagnosis accuracy with 71% sensitivity and an 11% decrease in specificity, which were statistically significant (*p* < 0.01)	The results were compared with six dental specialists (for grading) and 23 dentists. The model improved the dentists’ ability to detect dental caries (enamel-only proximal) significantly. It could be utilized by dentists as a supportive tool in preventive dentistry practice.
Zaorska et al., 2021 [57]	CNN	Predicting dental caries based on selected polymorphisms using an AI model	Datasets	95 patients	93% overall accuracy (*p* < 0.0001), 90.9–98.4% prediction accuracy, 90% sensitivity, 96% specificity, and 0.97 AUC (*p* < 0.0001)	A logistic regression model used. The AI model showed high accuracy in dental caries prediction. The knowledge of the status of potential risks can be useful in designing practices of oral hygiene and recommendations of dietary habits for patients.
Zheng et al., 2021 [58]	CNN	Evaluating and comparing 3 CNN models (VGG19, Inception V3, and ResNet18) for deep dental caries diagnosis	Radiographs	844 radiographs (717 for training and 127 for testing)	0.82 accuracy, 0.81 precision, 0.85 sensitivity, 0.82 specificity, and 0.89 AUC	The results were compared with experienced dentists, VGG19, and Inception V3. Among the three CNN models, ResNet18 displayed good performance. With regards to clinical parameters, the model showed an enhanced performance.
Mertens S et al., 2021 [16]	CNN	Detecting proximal dental caries using bitewing radiographs	Bitewing radiographs	140 (20 for testing) patients	0.89 ROC and 0.81 sensitivity (*p* < 0.05)	The results were compared with expert dentists. Dentists who used an AI model showed a significantly better performance than those who did not. Dentist diagnostic accuracy could be enhanced through this model.
Vinayahalingam et al., 2021 [59]	CNN	Evaluating MobileNet V2 used for the classification of dental caries on panoramic radiographs	Panoramic radiographs	500 radiographs (320 for training, 80 for validating, and 100 for testing)	0.87 accuracy, 0.86 sensitivity, 0.88 specificity, 0.90 AUC, and 0.86 F1 score	The results were compared with a reference standard. The model showed good performance in dental caries detection in 3rd molars. The model could initiate the development of a model that would help clinicians in making the decision of removing 3rd molars.
Lee et al., 2021 [60]	CNN	Evaluating U-Net (deep CNN) models for detecting dental caries on bitewing radiographs	Bitewing radiographs	304 for training and 50 for testing	63.29% precision, 65.02% recall, and 64.14% F1 score	The results were compared with three expert dentists. The model demonstrated sufficient performance in dental caries detection. Through this model, clinicians could detect dental caries more accurately.
Mao et al., 2021 [61]	CNN	Identifying dental caries and restorations in bitewing radiographs	Bitewing radiographs	278 images (70% for training and 30% for testing)	95.56% accuracy for restoration judgment and 90.3% accuracy for dental caries judgment	The results were compared with reference models (GoogleNet, Vgg19, and ResNet50). The AlexNet model displayed high accuracy than other models. Through this model, dentists would be able to make better decisions and treatment plans.
Huang et al., 2021 [62]	CNN	Detecting dental caries using AI models: AlexNet, ResNet-152, ResNext-101, VGG-16, and Xception	Micro-CT and OCT images	748 2D cross-sectional images (599, training; 149, testing)	ResNet-152 showed the highest rates of accuracy (95.21%), sensitivity (98.85%), and specificity (89.83%) with 93.48% PPV and 98.15% NPV values	The results were compared with five clinicians. The ResNet-152 CNN models distinguished pathological tooth structures better than clinicians. These models would help clinicians to provide more accurate diagnoses for patients.
Cantu et al., 2020 [17]	CNN	Identifying carious lesions	Bitewing radiographs	3293 for training and 252 for testing	0.80 accuracy, 0.75 sensitivity, and 0.80 specificity	The results were compared with four experienced individual dentists. The trained neural network had higher precision (0.80 vs. 0.71), specificity (0.83 vs. 0.9) and sensitivity (0.75 vs. 0.36) values than individual dentists. The model can assist dentists, more so in detecting initial caries lesions.
Schwendicke et al., 2020 [63]	CNN	Detection of dental caries in near-infrared-light transillumination (NILT) images	NILT images	226 images of extracted teeth	0.74 mean AUC, 0.59 sensitivity, 0.76 specificity, 0.63 PP, and 0.73 NPV	The results were compared with two expert dentists. The models detected dental caries satisfactorily. They would be relevant in environments such as care homes, rural outpost centers, and schools.
Casalegno et al., 2019 [23]	CNN	Automatically detecting and localizing dental caries in NILT images	NILT images	217 grayscale images and 185 for training	72.7% mean IOU score on a 5-class segmentation task, 49.5% and 49.0% IOU scores, and 83.6% and 85.6% ROC curves for proximal and occlusal carious lesions, respectively	The results were compared with experts and a reference deep neural network model. The DL approach increased the accuracy and speed of caries detection. The model could support dentists through the provision of high-throughput diagnostic help and the improvement of patient outcomes.
Ekert et al., 2019 [30]	CNN	AI system for detecting apical lesions	Panoramic radiographs (OPGs)	2001 panoramic radiographs	0.85 AUC, 0.65 sensitivity, and 0.8 specificity	The results were compared with six dentists. The model was effective in finding apical lesions in panoramic dental radiographs. It can assist dentists in detecting apical lesions more accurately in panoramic dental radiographs.
Choi et al., 2018 [64]	CNN	Detecting proximal dental caries	Periapical radiographs	475 periapical radiographs	0.74 F1max with 0.88 false positives	The results were compared with experienced dentists and a naïve CNN method as a reference model. The model proved superior to the naïve CNN model. Proximal dental caries were successfully detected by this model.
Imangaliyev et al., 2016 [65]	CNN	Automated classification model (CNN model) for red fluorescent dental plaque images	Quantitative light-induced fluorescence images	427 images	F1 score of 0.75 ± 0.05 on the test dataset	The results were compared with reference models. The model had higher prediction accuracy than the other models. The model benefitted directly from the images‘ multi-channel representation, hence improving the performance when the three color channels were utilized.
Dayi et al., 2023 [66]	DCDN	Evaluating the performance (diagnostic) of an AI system based on deep learning (Dental Caries Detection Network) for the segmentation of occlusal, cervical, and proximal caries lesions using panoramic radiographs	Panoramic radiographs	504 anonymous panoramic radiographs (75% for training and 25% for testing)	62.79% average F1 score and 15.69% highest average F1 score in state-of-the-art segmentation models	The results were compared with reference models. The system detected occlusal and proximal caries successfully but demonstrated weak performance in the detection of cervical caries. These systems could become common in dental clinics, as they increase the success rate in diagnosis and treatment, while also assisting dentists.
Lee et al., 2018 [26]	DCNN	Identifying dental caries in periapical radiographs	Periapical radiographs	2400 photos for training and validation and 600 for testing	82% diagnostic accuracy for both models, 88% for molars, and 89% for premolars	Dental caries were successfully detected. This model is potentially useful for detecting and diagnosing dental caries.
